# Luteolin enhances antitumor immunity of B7-H3-targeted bispecific natural killer cell engagers against non-small cell lung cancer

**DOI:** 10.7150/ijbs.125834

**Published:** 2026-03-25

**Authors:** Shuo Yang, Zhenfeng Zhang, Xi He, Zilu Ouyang, Shuzhen Tang, Manshan Liang, Zhuowen Chen, Yinghua Liu, Yang Huang, Yeneng Dai, Shengfeng Huang, Bihui Cao, Zhe-Sheng Chen, Xiyong Yu, Qi Zhao

**Affiliations:** 1 Guangzhou Municipal and Guangdong Provincial Key Laboratory of Molecular Target & Clinical Pharmacology, the NMPA and State Key Laboratory of Respiratory Disease, School of Pharmaceutical Sciences, Guangzhou Medical University, Guangzhou 511436, China.; 2 Department of Radiology, Translational Provincial Education Department Key Laboratory of Nano-Immunoregulation Tumor Microenvironment, the Second Affiliated Hospital of Guangzhou Medical University, Guangzhou, China.; 3 Department of Pharmaceutical Sciences, College of Pharmacy and Health Sciences, St. John's University, New York, NY, USA.; 4 Cancer Centre, Institute of Translational Medicine, Department of Biomedical Sciences, Faculty of Health Sciences, University of Macau, Taipa, Macau SAR, China.; 5 MoE Frontiers Science Center for Precision Oncology, University of Macau, Taipa, Macau SAR, China.

**Keywords:** B7-H3, luteolin, bispecific antibody, NK cell, non-small cell lung cancer, combination strategy

## Abstract

Non-small-cell lung cancer (NSCLC) is the predominant form of lung cancer and the leading cause of cancer-related mortality worldwide, largely due to its aggressive progression and resistance to current therapies. B7-H3 has emerged as a novel immunotherapeutic target worthy of investigation. Luteolin, a flavonoid polyphenolic compound abundantly present in vegetables and herbs, has demonstrated significant anti-tumor effects in various cancer types. However, its therapeutic mechanism of action in NSCLC remains poorly understood. This study aimed to examine the combined effects of luteolin and B7-H3-targeted immunotherapy in NSCLC. The results demonstrated that luteolin suppressed NSCLC cell proliferation in a dose-dependent manner and exhibited enhanced combined effects with B7-H3 inhibition, while also promoting apoptosis. This combination strategy produced significant inhibitory effects both *in vitro* and *in vivo*. A B7-H3-targeted bispecific killer cell engager (BiKE) was constructed, and antibody-dependent cell-mediated cytotoxicity (ADCC) was measured to evaluate its combined effect with luteolin. The B7-H3-targeted BiKE showed superior activity when combined with luteolin compared to either treatment of luteolin or BiKE alone. Our findings not only identify B7-H3 as a promising therapeutic target for NSCLC but also suggest luteolin as a potential anticancer adjuvant. The rationally designed combination strategy presented here may enhance existing treatment paradigms for NSCLC.

## Introduction

The role of immunotherapy in cancer was underappreciated for decades because tumors could severely interfere with immune responses by triggering negative regulatory pathways (also called immune checkpoints) related to immunological homeostasis or by adopting features that enable immune evasion 1. Programmed cell death 1 (PD-1) is a key regulator of T-cell signaling that is often exploited by tumors to evade immune surveillance. Its binding to PD-L1 inhibits antitumor immunity via immune checkpoint pathways. Several anti-PD-1/PD-L1 antibodies have consequently been developed for clinical use 2-4. While effective in some patients, limited benefit in others due to primary or acquired resistance indicates that PD-1 signaling is not the sole factor in immune evasion. Therefore, identifying new therapeutic targets remains crucial 5,6.

One such promising target is B7-H3 (CD276), a member of the B7 immune checkpoint family. It is highly expressed on cancer cells and tumor-infiltrating immune cells, enabling tumors to escape cytotoxic T-cell and natural killer (NK) cell-mediated destruction 7,8. Recently, B7-H3 has emerged as a valuable target for antibody-based immunotherapy due to its overexpression in diverse malignancies and cancer stem cells, its high prevalence (observed in ~60% of 25,000 tumor samples) with limited heterogeneity across cancer types, and its minimal expression in normal tissues 9,10. Evidence indicates that B7-H3 promotes tumor proliferation, metastasis, and therapy resistance, which correlates with poor clinical outcomes, making it an attractive therapeutic target 11. Furthermore, B7-H3 inhibits T-cell activation and proliferation, facilitates immune evasion, and modulates tumor progression through multiple signaling pathways 12. Consequently, B7-H3 presents an ideal target for innovative immunotherapies, such as bispecific antibodies (BiAbs). This therapeutic modality targets two antigens by binding to distinct recognition sites, thereby connect immune cells with tumor cells to induce cytotoxicity and demonstrating broad potential for cancer immunotherapy 13. Currently, a number of BiAb drugs have already been launched and put into use, showing significant therapeutic potential. We have previously demonstrated that bispecific killer cell engagers (BiKEs) targeting B7-H3 and CD16a (FcγRIIIa) boosted NK cell-mediated antitumor activity regardless of target antigen density on tumors 14.

Building on the promise of engineered immunotherapies, research is also exploring the potential of natural compounds to augment such responses. Among them, luteolin, a natural flavonoid, exhibits broad pharmacological effects, including anti-hyperlipidemic, anti-anaphylactic, anti-arthritic, and anti-inflammatory properties, which support its potential for clinical application 15,16. Notably, luteolin and its derivative apigenin enhance immunotherapy in KRAS (Kirsten rat sarcoma viral antigen)-mutant NSCLC by inhibiting proliferation, inducing apoptosis, and downregulating PD-L1 expression, thereby augmenting immune responses. Moreover, combining PD-1 blockade with luteolin has shown a synergistic effect, highlighting its clinical potential for KRAS-mutant NSCLC 17. Wu *et al.* 18 demonstrated that luteolin suppressed the proliferation and metastasis of androgen receptor-positive triple-negative breast cancer (TNBC) by downregulating matrix metalloprotease 9 (MMP9) expression via reducing Akt/mTOR-induced H3K27Ac and H3K56Ac levels. Osimertinib, a third-generation EGFR tyrosine kinase inhibitor used to treat NSCLC with specific EGFR mutations (including T790M)), selectively inhibits mutant EGFR signaling while sparing wild-type EGFR, thereby reducing side effects. It is a commonly applied therapy for eligible NSCLC patients. Luteolin has been shown synergize with osimertinib to overcome mesenchymal-epithelial transition factor (MET) amplification and overactivation-induced acquired resistance by inhibiting the hepatocyte growth factor (HGF)-MET-Akt pathway, suggesting a promising therapeutic strategy for NSCLC patients with osimertinib resistance 19.

In this study, we report, for the first time, the combined antitumor efficacy of luteolin with a B7-H3-targeted BiKE in NSCLC models. We demonstrate that both anti-B7-H3 therapy and luteolin significantly suppress NSCLC proliferation by modulating reactive oxygen species (ROS) levels and promoting apoptosis. Notably, the novel combination of luteolin and B7-H3-BiKE exhibited markedly enhanced NK cell activation and therapeutic effects, suggesting a cooperative strategy that has not been previously explored. These findings establish a new combinatorial paradigm with potential transformative significance for NSCLC treatment.

## Materials and Methods

### Reagents and cell culture

B7-H3 small interfering RNA (siRNA) was synthesized by RIBOBIO (Guangzhou, China), and the B7-H3 overexpression plasmid was obtained from Tsingke (Beijing, China). Luteolin was purchased from ACMEC (Shanghai, China). Human NSCLC cell lines A549, HCC827, PC9, and H1703 were used in this study. A549 cells were obtained from Guangzhou Jiayan Biotechnology Co., Ltd., while HCC827 and PC9 cells were kindly provided by Professor Zhang Zhenfeng (The Second Affiliated Hospital of Guangzhou Medical University), and H1703 cells were donated by Professor Lei Xueping (School of Pharmacy, Guangzhou Medical University). All cells were cultured in RPMI-1640 medium (Gibco, Grand Island, NY) supplemented with 10% fetal bovine serum (FBS) (Gibco, Grand Island, NY) and 1% penicillin-streptomycin at 37°C in a 5% CO_2_ incubator.

### Bulk RNA sequencing

Cells were cultured in 100 mm plates and divided into two groups: control group (control) and B7-H3 knockdown group (siCD276). After 24 h, total RNA was extracted using TRIzol reagent (#15596026, Invitrogen), flash-frozen in liquid nitrogen, and then subjected to transcriptome sequencing (LC-Bio Technology Co., Ltd., Hangzhou, China). Bioinformatics analysis was performed using R Statistical Software (v4.1.2), and differentially expressed genes (DEGs) were identified using the DESeq2 package.

### Kaplan-Meier survival analysis

Prognostic significance of B7-H3 in NSCLC was assessed using the Kaplan-Meier Plotter (http://kmplot.com/analysis/), which integrates gene expression and clinical data. Overall survival (OS), first-progression survival (FPS), and post-progression survival (PPS) was evaluated, with hazard ratios (HR), 95% confidence intervals (CI), and p-values of the log-rank calculated 20.

### Analysis of TCGA data in lung adenocarcinoma and squamous cell carcinoma

We obtained TCGA gene expression profiles and clinical data for lung adenocarcinoma (LUAD) and lung squamous cell carcinoma (LUSC) cohorts using the easyTCGA R package. Data analysis was performed with R software.

### CCK-8 assay

Cell viability was assessed using the Cell Counting Kit-8 (CCK-8; DOJINDO, Tokyo, Japan). Briefly, cells were seeded in 96-well plates at a density of 5×10³ cells per well and allowed to adhere overnight. Following the respective treatments, the CCK-8 reagent was added to each well, and the plates were incubated at 37 °C for an additional 2 h. The absorbance at 450 nm was then measured using a microplate reader. Cell viability was calculated and compared to the untreated control group.

### Colony formation assay

The clonogenic ability of cells was evaluated using a colony formation assay. Cells were seeded at a low density (1,000 cells per well) in 12-well plates and cultured under standard conditions for 8 days to allow for colony development. Subsequently, the resulting colonies were fixed with 4% paraformaldehyde for 20 min and stained with 0.1% crystal violet solution for 30 min at room temperature. After gently washing with phosphate-buffered saline (PBS) to remove excess dye, colonies were counted, and the relative colony formation ability was calculated for each group.

### siRNA and plasmid transfection

To investigate the functional role of B7-H3 in cellular processes, targeted silencing and overexpression of B7-H3 were performed using RNA interference and plasmid transfection approaches. Specifically, a custom B7-H3 siRNA (target sequence: “CAACGAGCAGGGCTTGTTT”) and a B7-H3 overexpression plasmid were transfected into cells using Lipofectamine 3000 transfection reagent (Invitrogen), following the manufacturer's instructions. Cells were seeded in appropriate culture plates and allowed to adhere overnight prior to transfection. The transfection complex was prepared by mixing the siRNA or plasmid DNA with Lipofectamine 3000 reagent in Opti-MEM medium, followed by incubation and subsequent addition to the cells. After 24 h, transfection efficiency was validated through Western blot analysis.

### Wound-healing assay

Cell migration capability was evaluated using a wound-healing assay. Briefly, cells were seeded into six-well plates and cultured overnight to form a confluent monolayer. After pretreatment with luteolin or modulation of B7-H3 expression via siRNA or plasmid transfection, a uniform scratch was created in the cell monolayer using a sterile 200 μL pipette tip when the cell density reached approximately 95%. The plates were then gently washed to remove detached cells and replenished with fresh medium. Wound closure was monitored at different time points under an optical microscope, and images of the scratch area were captured at each time point. The migration rate and wound healing percentage were quantified by measuring the change in scratch width using ImageJ software, with at least three independent biological replicates performed per experimental condition to ensure statistical reliability.

### Migration assay

Cell migratory capacity was further assessed using a transwell migration assay. Transwell inserts with an 8-μm pore size (Corning, NY, USA) were employed for this assay. In each experiment, the upper chamber was coated with serum-free medium containing 1×10^5^ cells subjected to the indicated treatments, such as luteolin exposure or B7-H3 modulation. The lower chamber was filled with 600 μL of complete medium containing 30% FBS. After 24 h of incubation at 37 °C in a 5% CO_2_ atmosphere, non-migrated cells on the upper surface of the membrane were carefully removed. The migrated cells on the lower surface were fixed with 4% paraformaldehyde, stained with 0.1% crystal violet solution, and visualized under a microscope. The number of migrated cells was quantified from photographed images using ImageJ software.

### Production and verification of the B7-H3/CD16 protein

The B7-H3/CD16 protein was engineered by fusing two single-chain variable fragments (scFvs). The variable heavy (VH) and light (VL) chains of the humanized anti-B7-H3 antibody (8H9) and the anti-CD16 antibody (3G8) were connected individually via a (GGGGS)_3_ linker to generate respective scFvs. These scFv genes were then assembled into a single construct of B7-H3/CD16 protein using an additional (GGGGS)_3_ linker. The resulting sequence was cloned into a pSecTag B expression vector for production. Specifically, the recombinant proteins were expressed in Freestyle 293-F cells via transfection. The resulting BiAb was then purified from the culture supernatant by affinity chromatography using Ni-NTA agarose beads (Qiagen), employing a previously described method 10. The expressed B7-H3/CD16 BiAb was separated by sodium dodecyl sulfate-polyacrylamide gel electrophoresis (SDS-PAGE). The gel was then stained with coomassie brilliant blue (CBB) overnight. The image was captured after destaining.

### Enzyme-linked immunosorbent assay (ELISA)

The binding specificity and affinity of the purified B7-H3/CD16 BiAb to its target antigens were evaluated using enzyme-linked immunosorbent assay (ELISA). Following the manufacturer's protocol, 96-well plates were coated with either soluble 4Ig-B7-H3 or CD16a proteins and incubated overnight at 4 °C. After blocking with 5% skim milk to prevent nonspecific binding, the B7-H3/CD16 BiAb was added at varying concentrations and incubated for 1 h at room temperature. Following washing, an anti-His-tag antibody (48140-1, SAB) was applied, and the signal was developed using a tetramethylbenzidine (TMB) substrate. The reaction was then stopped, and the absorbance was measured at 450 nm using a microplate reader. Each experiment included appropriate controls and was performed in triplicate.

### Cytotoxicity assay

The cytotoxicity of BiKE combined with luteolin was assessed following a previously described protocol 14. Peripheral blood mononuclear cells (PBMCs) were employed to model the physiological immune microenvironment. This allows us to not only measure direct NK cell cytotoxicity but also to uncover potential contributions from other immune cells that may enhance the overall anti-tumor response. Briefly, calcein-AM-labeled NSCLC target cells were co-cultured with PBMCs as effector cells at an effector-to-target (E:T) ratio of 10:1, and treated with B7-H3/CD16 BiAb (10 nM) and/or luteolin (10 nM) for 4 h at 37°C. After incubation, cells were washed 2-3 times with PBS and examined under a fluorescence microscope in serum-free medium. Spontaneous and maximum release controls were included by incubating target cells alone or with lysis buffer (Triton X-100), respectively, and the number of stained cells was used to indicate the efficacy of cytotoxicity.

### Flow cytometry

The surface expression of B7-H3 on NSCLC cells was analyzed using PE-conjugated anti-human CD276 (B7-H3) antibody (BioLegend). For apoptosis analysis, cells were washed twice with PBS and stained with Annexin V-FITC (fluorescein isothiocyanate) and PI (propidium iodide) using the Annexin V-FITC Apoptosis Detection Kit (Dojindo, Tokyo, Japan). Apoptosis was then quantified by flow cytometry using a CytoFLEX instrument (Beckman Coulter, USA). The intracellular ROS was measured using 2,7-dichlorofluorescin diacetate (DCFH-DA) as a probe. A549 and HCC827 cells were harvested and incubated with varying concentrations of luteolin for 24 h. Then, cells were resuspended and incubated with the probe for 15 min and then washed once with PBS. Regarding the activation of NK cells, PBMCs were used as effector cells and A549 cells as target cells with (E:T) ratio of 10:1, with B7-H3/CD16 BiAb at a concentration of 10 nM were administered either alone or in combination with luteolin. After incubation for 4 h, CD56 (MultiSciences) and CD16 (BioLegend) were detected to indicate the activation status of NK cells. Fluorescence intensity was measured using the CytoFLEX instrument (Beckman Coulter, USA).

### Western blotting

Equal amounts of protein were separated by SDS-PAGE and transferred to polyvinylidene difluoride (PVDF) membranes (Millipore). The membranes were then blocked with 5% skim milk for 1 h and incubated overnight with primary antibodies at 4 °C. Subsequently, the membranes were incubated with secondary antibodies for 1.5 h at room temperature. Protein bands were visualized and analyzed using the ChemiDoc XRS+ System. The following primary antibodies were used: anti-B7-H3 (14058S), anti-phospho-AKT (4060T), anti-rabbit IgG HRP-linked antibody (7074S), and anti-mouse IgG HRP-linked antibody (7076S) from Cell Signaling Technology; anti-AKT antibody (10176-2-AP) from proteintech; and anti-Tublin (ab6046) and anti-GAPDH (ab181602) from Abcam.

### Animal experiments

All animal experimental procedures were conducted in accordance with ethical guidelines and approved by Guangzhou Medical University Laboratory Animal Center. Four- to six-week-old BALB/c nude mice were obtained from Guangdong Provincial Medical Laboratory Animal Center (Foshan, China) and maintained under specific-pathogen-free (SPF) conditions. Briefly, A549 cells (2×10^6^ per mouse) were subcutaneously injected into each mouse. Animals were randomized (n = 5) when tumor volumes reached approximately 100 mm³. To evaluate the therapeutic efficacy of B7-H3 siRNA and luteolin *in vivo*, mice in each group received twice-weekly intraperitoneal injections, beginning on day 0, with one of the follwing: PBS (control), luteolin (50 mg/kg), B7-H3 siRNA (1 nmol/mouse), or a combination of luteolin and B7-H3 siRNA. Tumor diameters were measured every three days. After approximately one month of treatment, mice were euthanized and tumors were excised for measurement.

For assessing the therapeutic effects of B7-H3/CD16 BiKE and luteolin, HCC827 cells (2×10^6^ per mouse) were subcutaneously inoculated into NOD-SCID mice. Four- to six-week-old NOD-SCID mice were obtained from Slack Laboratory Animal Co. (Shanghai, China) and maintained under SPF conditions. The B7-H3/CD16 BiAb was administered intravenously twice weekly for a total of four doses. Treatment groups received BiKE with or without luteolin (50 mg/kg), with PBMCs from healthy donors (5×10^6^ cells/mouse) serving as effector cells via intravenous injection starting from day 0. Tumor growth was monitored every three days using digital calipers, with tumor volume calculated using the formula: Volume = 0.5 × Length × Width².

### Immunohistochemistry

Paraffin sections were deparaffinized in xylene, rehydrated through a graded ethanol series, and rinsed in PBS. For antigen retrieval, slides were microwaved in retrieval buffer for 10 min, cooled in running water, and rinsed with PBS. Endogenous peroxidase was blocked with 3% H_2_O_2_-methanol for 10 min at RT, followed by PBS washes. After blocking with 1% BSA for 20 min, primary antibody (Rabbit-anti-Ki67, ABCAM, ab16667) was applied and incubated overnight at 4°C. Slides were washed, then incubated with goat anti-rabbit/mouse polymer secondary antibody for 20 min. DAB was used for color development, which was monitored microscopically and stopped by rinsing with tap water. Counterstaining was performed with hematoxylin, followed by dehydration through graded alcohols and xylene, and mounted with a neutral mounting medium. Protein expression was evaluated under an optical microscope and photographed at 400× magnification.

### Immunofluorescence

Deparaffinized sections were rehydrated through xylene and a graded ethanol series, followed by PBS rinses. Antigen retrieval was performed via microwave heating in citrate buffer and cooling naturally. Endogenous peroxidase was blocked with 3% H_2_O_2_-methanol, followed by PBS washes. Sections were incubated with 1% BSA for 20 min, followed by primary antibody (rabbit anti-CD31, ABCAM, ab182981) at 37 °C for 2 h, and an FITC-conjugated secondary antibody (Jackson ImmunoResearch, 111-095-003) at 37 °C for 1 h, protected from light. After PBS washes, nuclei were counterstained with DAPI, and slides were mounted with anti-fade medium before imaging using a digital slide scanner.

### Statistical analysis

All data shown are representative of at least three independent experiments and are expressed as mean ± standard deviation (SD). Statistical significance was determined using Student's t-test for comparisons between two groups and one-way ANOVA for comparisons among more than two groups. All statistical tests were analyzed using GraphPad PRISM software (version 9.0; La Jolla, CA, USA). *P* < 0.05 indicated a significant difference.

## Results

### Analysis of B7-H3 expression profiles in NSCLC patients and cell lines

**Figure 1:**
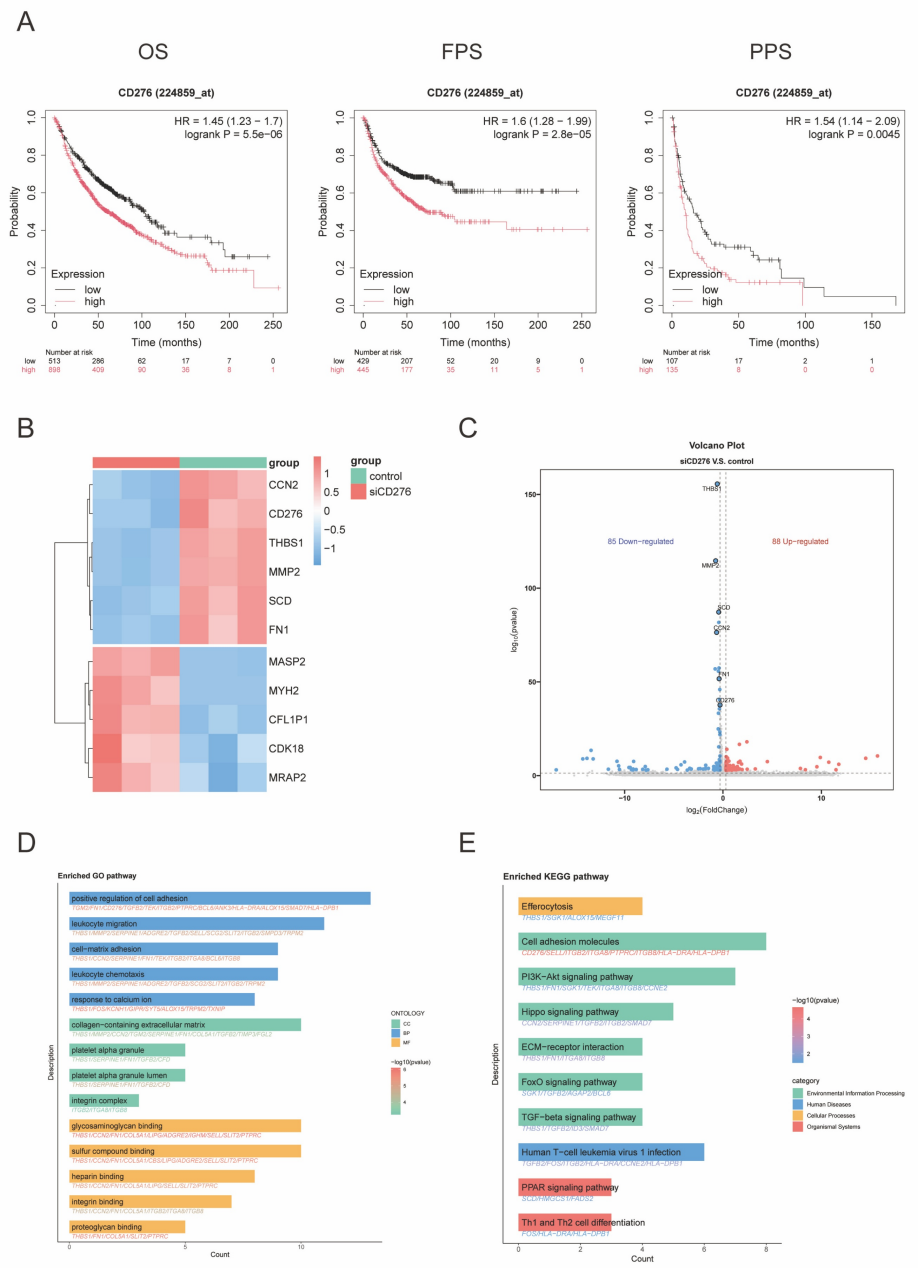
** Identification of B7-H3 related variations in NSCLC. (A)** Overall survival (OS), post-progression survival (PPS), and first-progression survival (FPS) for patients with low or high levels of B7-H3 expression by Kaplan-Meier analysis. The data were generated from the TCGA database. **(B)** Heatmap of genes differentially expressed upon B7-H3 knockdown.** (C)** Volcano plot of differentially expressed genes; blue indicates downregulated genes and red denotes upregulated genes. Gene Ontology (GO) **(D)** and Kyoto Encyclopedia of Genes and Genomes (KEGG) pathway analyses **(E)** were performed to investigate the biological functions and pathways associated with B7-H3-mediated differential gene expression.

Building upon our previous findings demonstrating broad B7-H3 expression across various tumors 14, we further investigated its expression pattern and clinical significance in NSCLC. Our analysis of the combined LUAD and LUSC dataset revealed distinct CD276 (B7-H3) expression profiles. Specifically, B7-H3 mRNA levels were significantly elevated in tumor tissues compared to normal counterparts. Furthermore, a progressive increase in expression was observed with disease advancement, as evidenced by significantly higher levels in stage II than in stage I. Intriguingly, significant differences in B7-H3 expression were also identified between different genders, suggesting a potential dimension for further exploration (Figure S1A-C). Given this association with tumor progression, we next evaluated its prognostic value. Kaplan-Meier survival curve analysis revealed that patients with relatively high B7-H3 expression predicted poor survival (Figure 1A). Flow cytometry confirmed high B7-H3 surface expression in a panel of NSCLC cell lines (Figure S2A), supporting its potential as a therapeutic target.

To explore the potential mechanisms through which B7-H3 influences NSCLC prognosis, we performed transcriptome sequencing following B7-H3 knockdown. Differential gene expression analysis was visualized in a heatmap (Figure 1B) and a volcano plot (Figure 1C), which illustrated significantly upregulated and downregulated genes and helped identify key targets for further investigation. To elucidate the functional mechanisms of B7-H3 in NSCLC, we performed Gene Ontology (GO) and Kyoto Encyclopedia of Genes and Genomes (KEGG) pathway analyses on the differentially expressed genes. These analyses revealed the most significantly enriched biological processes and signaling pathways (Figure 1D-E). Collectively, these results demonstrate that B7-H3 expression is not only highly conserved in NSCLC but also strongly associated with tumor progression and patient survival. Our findings underscore the critical role of B7-H3 in NSCLC pathogenesis and provide a rationale for developing targeted therapeutic strategies.

### Luteolin and anti-B7-H3 inhibit NSCLC cell viability and migration

**Figure 2:**
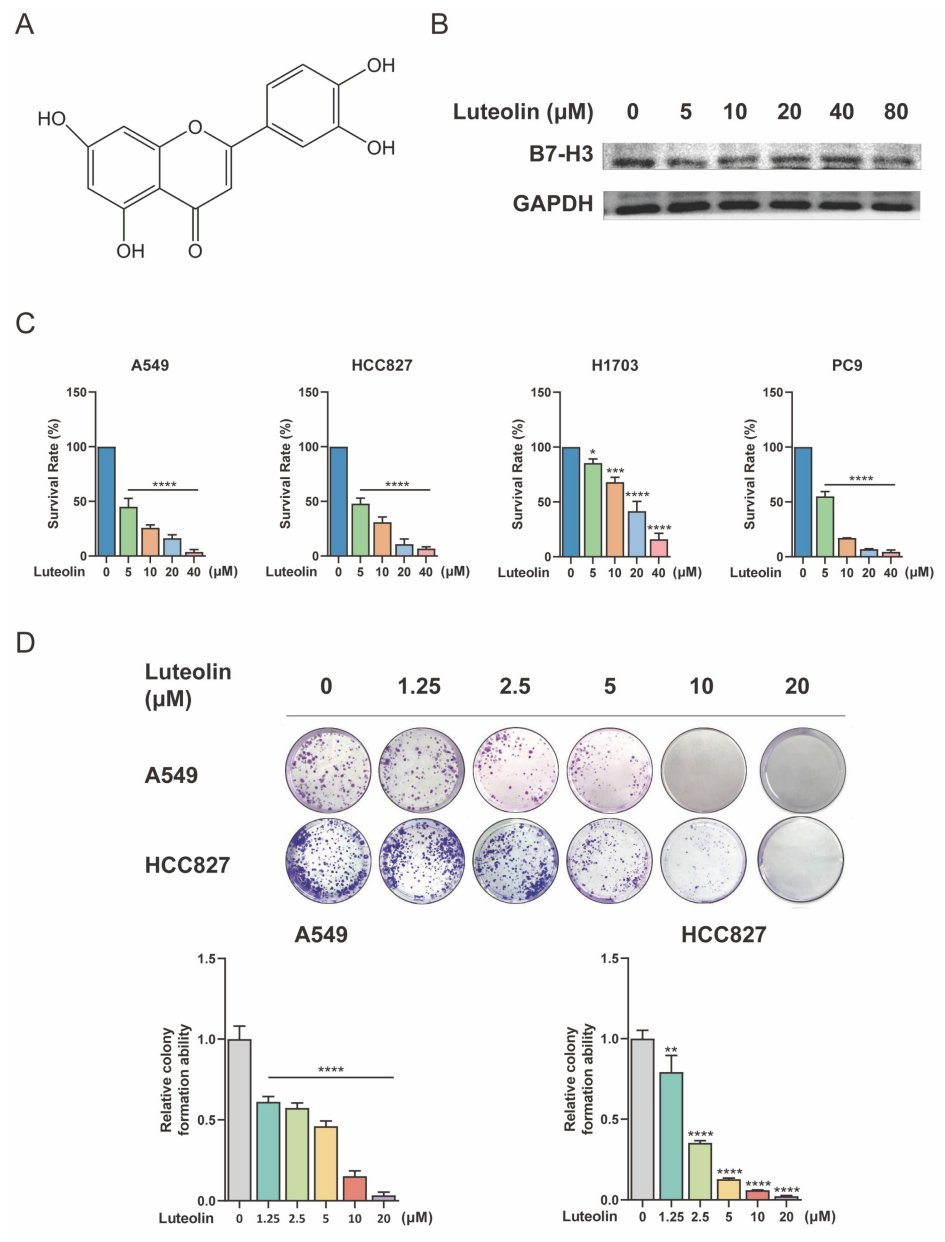
** Relationship between B7-H3 and luteolin and its effects on NSCLC cell viability. (A)** Chemical structure of luteolin. **(B)** The regulatory effects of luteolin on B7-H3 under different concentrations.** (C)** Cell viability was measured using the Cell Counting Kit-8 (CCK-8) assay after treating NSCLC cells with the indicated concentrations of luteolin for 24 h.** (D)** Colony formation ability was assessed by colony-formation assay. Data are shown as mean ± SD (n=3). *P < 0.05, **P < 0.01, ***P < 0.001, and ****P < 0.0001.

Given the therapeutic significance of B7-H3, we investigated whether the natural compound luteolin exerts its effect by modulating B7-H3 or its associated pathways. Luteolin (Figure 2A) has been reported to suppress NSCLC cell growth and induce apoptosis by modulating pathways such as ERK/MAPK 21. We treated A549 cells with different concentrations of luteolin and found that it had a certain regulatory effect on B7-H3 (Figure 2B). We then assessed the effect of luteolin on NSCLC cell growth. A549, HCC827, H1703, and PC9 cells were treated with increasing concentrations of luteolin. As shown in Figure 2C, luteolin significantly reduced cell viability in a concentration-dependent manner across all tested cell lines. Specifically, treatment with 5, 10, 20, and 40 μM luteolin reduced the viability of A549 cells to 45.10%, 25.83%, 16.26%, and 3.72%, respectively. Similarly, under the same treatment conditions, the viability of HCC827 cells decreased to 47.88%, 30.95%, 10.86%, and 6.78%. H1703 cells appeared relatively less sensitive, with viability rates of 85.38%, 68.02%, 41.54%, and 15.98%. Similar phenomena have also been observed in PC9 cells, where viability dropped to 55.11%, 17.03%, 6.95%, and 4.39% at the respective concentrations. These data robustly demonstrate the potent anti-proliferative effect of luteolin on NSCLC cells. To further validate these findings, we examined the effect of luteolin on the clonogenic activity of A549 and HCC827 cells using a colony formation assay. Consistent with the viability results, luteolin inhibited the colony-forming ability of both cell lines in a concentration-dependent manner (Figure 2D).

**Figure 3:**
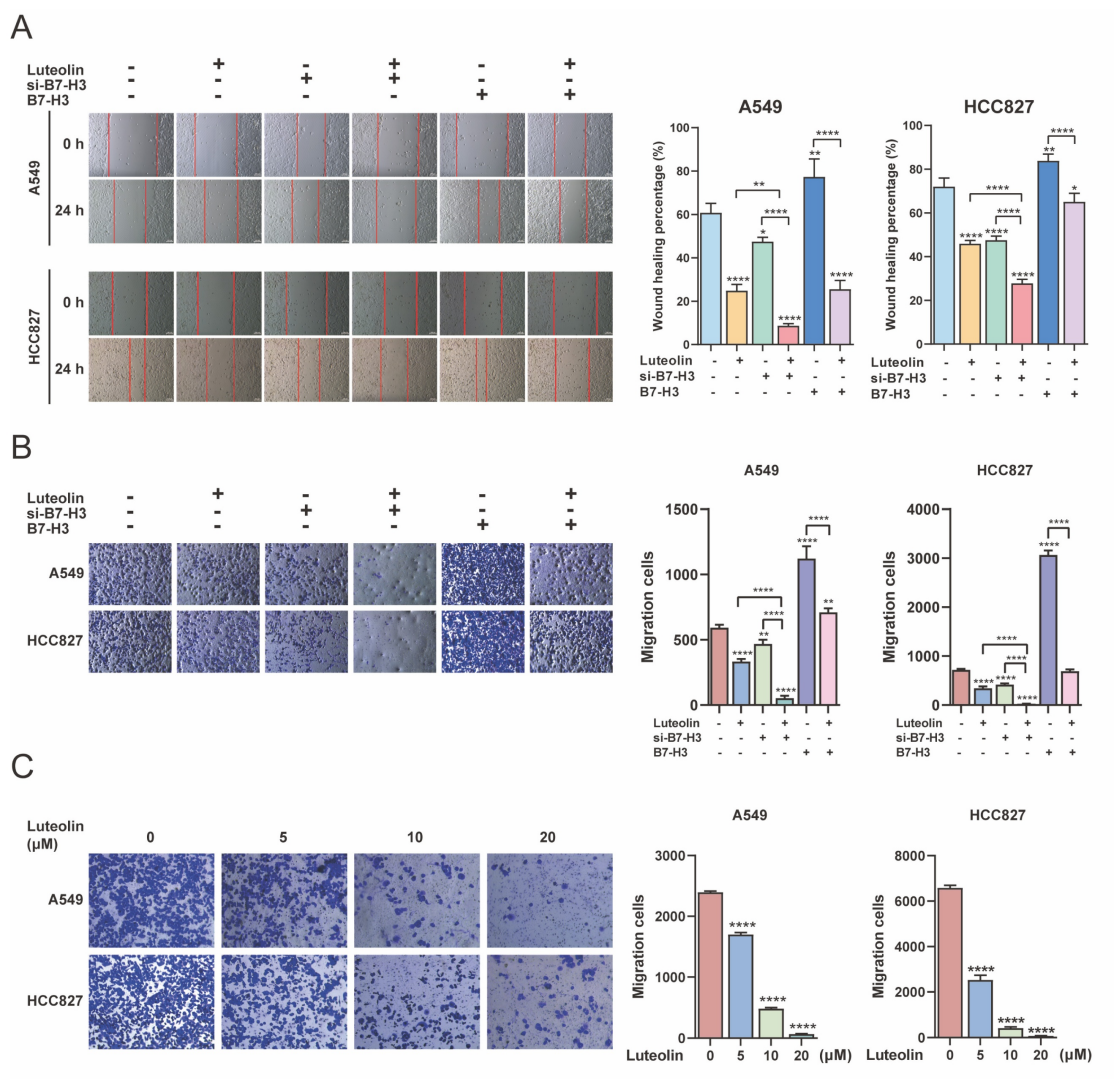
** Luteolin and B7-H3 downregulation inhibit the migration of NSCLC cells.** NSCLC cells were transfected with siB7-H3 or a B7-H3 overexpression plasmid, followed by treatment with or without luteolin (10 μM) for 24 h. **(A)** Cell migration was assessed using a wound-healing assay (scale bar=200 μm). **(B)** Cell migration was also evaluated by transwell assay following transfection.** (C)** The dose-dependent effect of luteolin on cell migration was assessed by transwell assay. *P < 0.05, **P < 0.01, and ****P < 0.0001.

To further examine the effect of luteolin in combination with B7-H3 targeting on NSCLC cell migration, we selected A549 and HCC827 cells for transfection with B7-H3 siRNA or a B7-H3 overexpression plasmid to establish knockdown (si-B7-H3) and overexpression (B7-H3) models. Successful transfection was confirmed (Figure S2B). Wound-healing assays demonstrated that si-B7-H3 significantly inhibited cell migration, while B7-H3 overexpression enhanced it. The inhibitory effect of luteolin on cell migration was further augmented by its combination with si-B7-H3. Furthermore, luteolin counteracted the migration-promoting effect induced by B7-H3 overexpression (Figure 3A). Meanwhile, luteolin alone inhibited NSCLC cell migration in a dose-dependent manner (Figure S3). Similarly, transwell migration assays showed that the combination of luteolin and si-B7-H3 had such effects, while luteolin alone inhibited NSCLC cell migration in a dose-dependent manner (Figure 3B-C). Collectively, our data suggest that B7-H3 functions as an oncogene and represents a potential therapeutic target in NSCLC, and that luteolin not only inhibits NSCLC cell migration but enhances the anti-migratory effect of B7-H3 knockdown.

### Luteolin induces apoptosis and modulates ROS levels

**Figure 4:**
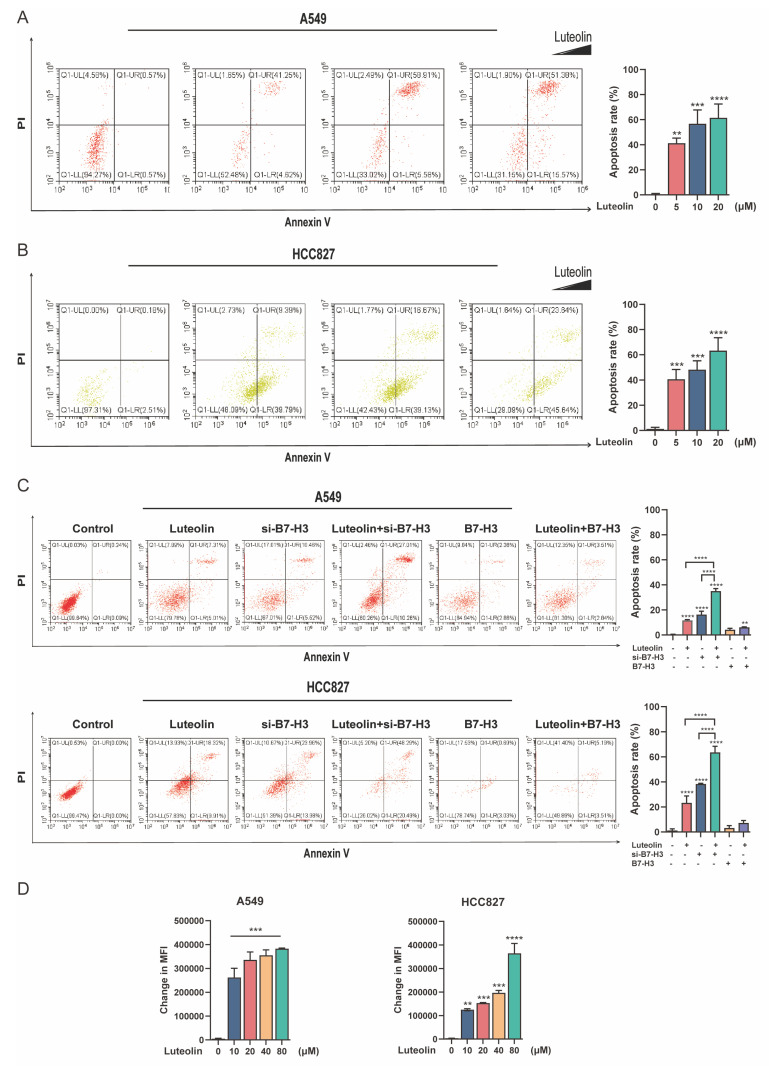
** Analysis of apoptosis and ROS levels by flow cytometry. (A)** Representative results and quantitative analysis of apoptosis in NSCLC cells treated with varying concentrations of luteolin. **(B)** Representative results and quantitative analysis of apoptosis in NSCLC cells with modulated B7-H3 expression, with or without luteolin (10 μM) treatment.** (C)** ROS levels following treatment with different concentrations of luteolin. **P < 0.01, ***P < 0.001, and ****P < 0.0001.

Apoptosis, a process of programmed cell death crucial for maintaining cellular homeostasis, plays a vital role in the pathophysiology of NSCLC 22. To explore the mechanism underlying the growth inhibition mediated by B7-H3 targeting and luteolin, we analyzed apoptosis by flow cytometry. We found that luteolin promoted apoptosis in A549 and HCC827 cells in a dose-dependent manner (Figure 4A). Quantitative analysis revealed that in A549 cells, treatment with 5, 10, and 20 μM luteolin induced apoptosis rates of 41.22%, 56.68%, and 61.42%, respectively. A similar trend was observed in HCC827 cells, where the apoptosis rates reached 40.69%, 48.29%, and 63.27% at the same concentration gradient. Subsequently, we investigated the combined effect of luteolin and B7-H3 modulation and found that the combination of luteolin and si-B7-H3 enhanced apoptosis.

Furthermore, luteolin was able to elevate apoptosis even in B7-H3-overexpressed cells (Figure 4B). Specifically, in A549 cells, the co-treatment of luteolin with si-B7-H3 resulted in the highest apoptosis rate (35.05%), substantially exceeding the rates observed with either treatment alone (11.61% for luteolin and 16.29% for si-B7-H3). This pattern was corroborated in HCC827 cells, where the combination of luteolin and si-B7-H3 elicited a profound apoptotic response (63.61%), markedly higher than that caused by the individual agents (23.31% for luteolin and 38.41% for si-B7-H3). Given the essential role of ROS in cell metabolism and apoptosis, we examined whether luteolin could alter ROS levels in NSCLC cells. Measurement of cellular ROS in A549 and HCC827 cells after luteolin treatment showed that luteolin increased ROS levels in a concentration-dependent manner (Figure 4C). Thus, luteolin likely promotes B7-H3-associated apoptosis, at least in part, through elevating cellular ROS.

### B7-H3 and luteolin affect tumor growth *in vivo*

**Figure 5:**
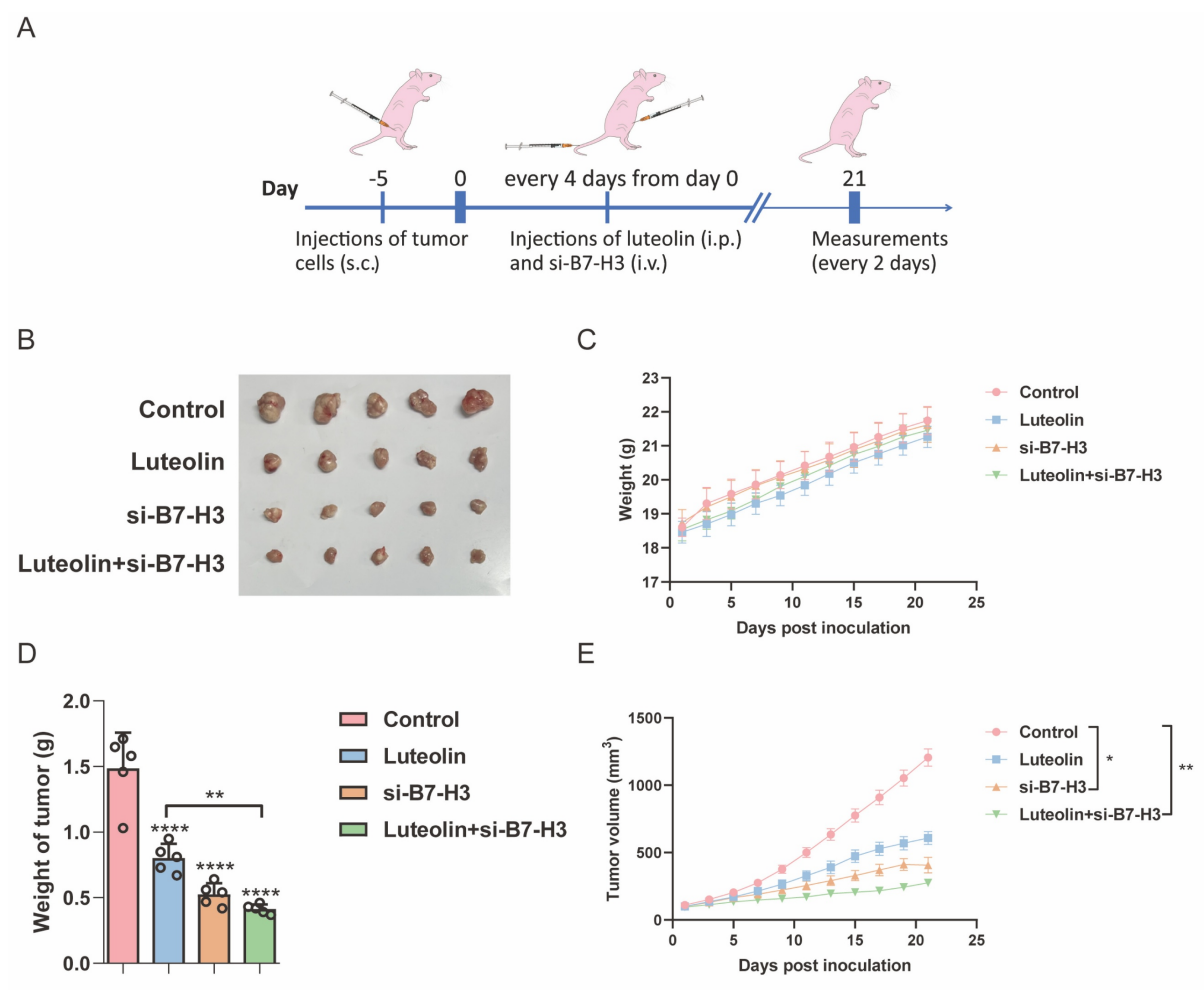
** Therapeutic effects of luteolin and si-B7-H3 on NSCLC xenografts. (A)** Schematic timeline of the treatment protocol in nude mice with subcutaneous A549 xenografts. Tumor volume was measured and calculated (n=5). **(B)** Mice were killed and the tumors were collected. **(C)** Body weight changes in each treatment group. **(D)** Comparison of tumor weights across groups. **(E)** Comparison of tumor volumes across groups. *P < 0.05, **P < 0.01, and ****P < 0.0001.

To investigate whether B7-H3 knockdown combined with luteolin treatment exhibits tumor-suppressive effects in NSCLC, we established A549 xenograft models to evaluate the efficacy of combination therapy *in vivo* (Figure 5A). When tumor volumes reached approximately 100 mm³, mice were randomly divided into four groups: control, luteolin alone, B7-H3 *in vivo* siRNA (RIBOBIO) alone, and luteolin combined with B7-H3 *in vivo* siRNA. We observed that both luteolin treatment and B7-H3 siRNA administration alone suppressed tumor growth, while the combination therapy resulted in significantly enhanced tumor suppression without causing significant changes in body weight among the groups (Figure 5B-E). These findings demonstrate that the combination of B7-H3 siRNA and luteolin exhibits superior antitumor efficacy in NSCLC xenograft models.

### Luteolin enhances the ADCC effect of B7-H3/CD16 BiKE

**Figure 6:**
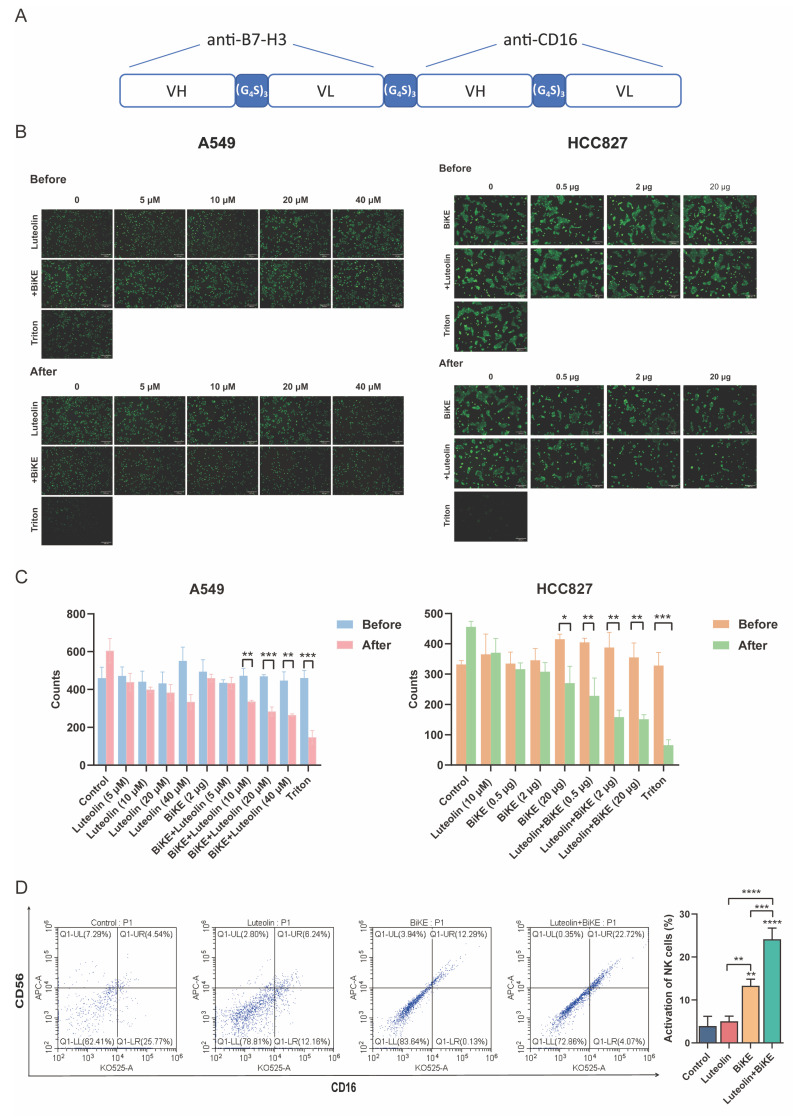
** Luteolin and B7-H3/CD16 BiKE induce NK cell-mediated antitumor effects. (A)** Schematic diagram of the B7-H3/CD16 BiKE construct. **(B, C)** Cytotoxic effects of B7-H3/CD16 BiKE with or without luteolin on NSCLC cells. PBMCs were used as effector cells. Representative results are shown (scale bar=200 μm). **(D)** Flow cytometry for detecting the activation level of NK cells. *P < 0.05, **P < 0.05, ***P < 0.001, and ****P < 0.0001.

Building on the promising efficacy of combinatorial B7-H3 targeting, we next developed a therapeutic modality designed to directly engaging immune cells. To this end, we constructed a B7-H3/CD16 BiKE by linking the anti-B7-H3 scFv and anti-CD16 scFv with a non-immunogenic (GGGGS)_3_ linker, followed by production and purification as previously described 14 (Figure 6A). The molecular weight of the BiKE protein was presented by SDS-PAGE (Figure S4A). ELISA demonstrated that the purified B7-H3/CD16 BiKE maintained binding capacity for both soluble 4Ig-B7-H3 and CD16 proteins (Figure S4B). To evaluate the antitumor efficacy, we performed antibody-dependent cellular cytotoxicity (ADCC) assays by co-incubating effector PBMCs with B7-H3-positive NSCLC cell lines (A549 and HCC827) in the presence of BiKE and/or luteolin (Figure 6B-C). The enhancement was demonstrated to be dose-dependent in two dimensions: increasing the concentration of luteolin at a fixed BiKE dose led to a progressive increase in tumor cell killing, and at a fixed luteolin concentration, escalating the BiKE dose also resulted in improved cytotoxic activity. This cell lysis is specifically mediated by NK cells within the PBMC population, initiated by the BiKE's dual-specificity, which cross-links B7-H3 on tumor cells with the CD16 activating receptor on NK cell. These findings demonstrated that the B7-H3/CD16 BiKE not only effectively mediated potent cytotoxicity against B7-H3-positive NSCLC cells, and this effect was significantly enhanced by luteolin in a dose-dependent manner. Furthermore, we also examined the activation effect of luteolin in combination with BiKE on NK cells. We conducted a flow cytometry experiment to detect the activation status of NK cells in PBMCs as the effector cells. The results showed that when luteolin combined with BiKE, it can effectively activate the NK cells in PBMCs, thereby enhancing their killing activity (Figure 6D).

### Luteolin and B7-H3/CD16 BiKE induce NK Cell-mediated antitumor efficacy

**Figure 7:**
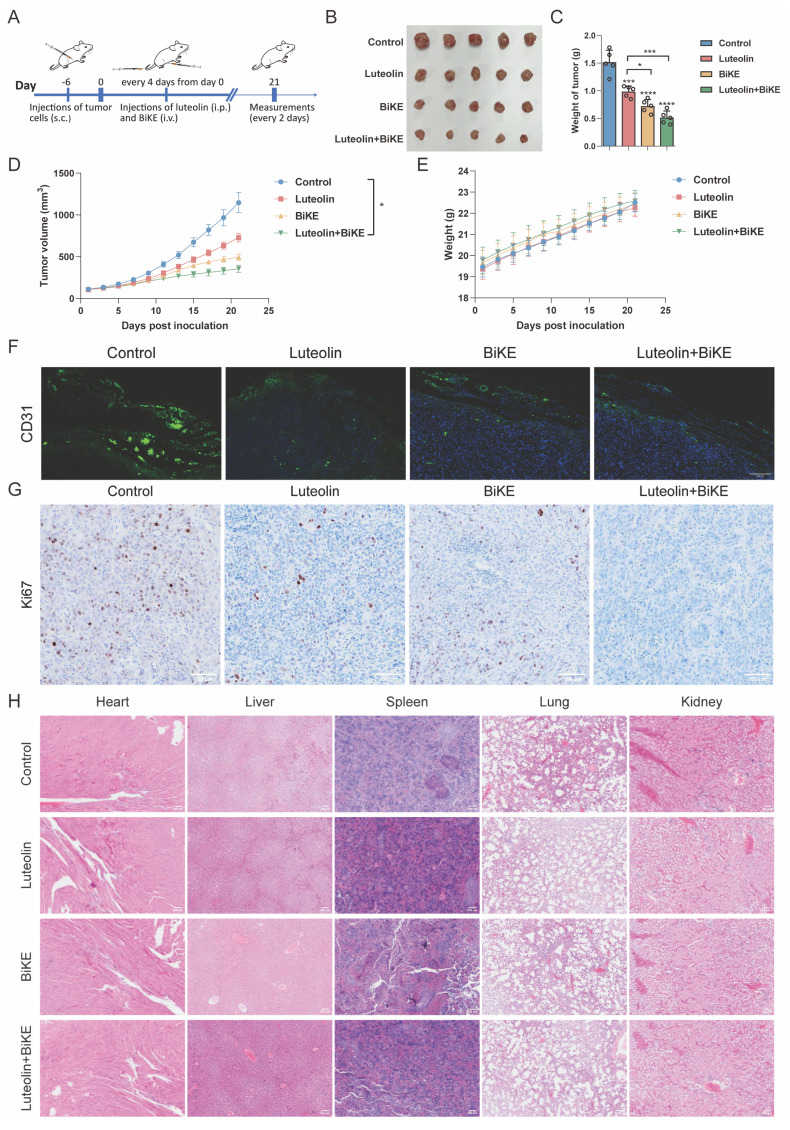
** Therapeutic effects of luteolin and B7-H3/CD16 BiKE in HCC827 xenografted mice. (A)** Treatment procedures for NOD/SCID mice subcutaneously inoculated with HCC827 cells. The BiKE antibody was administered intravenously (100 μg per dose) twice a week for two weeks. Human PBMCs (5×10⁶ cells per injection) were delivered intravenously twice (n=5). **(B)** Mice were killed and the tumors were collected. **(C)** Tumor weights of mice in each group (n=5). **(D)** Comparison of tumor growth curves among combination therapy, luteolin alone, BiKE alone, and control groups. **(E)** Body weight records of mice in each group. **(F)** Representative immunofluorescence images of CD31 in tumor samples (scale bar=200 μm).** (G)** Representative immunohistochemical images of Ki67 in tumor samples (scale bar=100 μm).** (H)** H&E-stained images of heart, liver, spleen, lung, and kidney tissues from each group (scale bar=200 μm). *P < 0.05, ***P < 0.001, and ****P < 0.0001.

We next evaluated the *in vivo* efficacy of the BiKE and luteolin using an HCC827 xenograft model in NOD/SCID mice (Figure 7A). Mice received intravenous injections of the BiKE twice weekly for two weeks. The combination therapy significantly reduced the tumor volumes compared with control group. No significant differences in body weight were observed among the four groups during the study (Figure 7B-E). Consistent with these findings, immunofluorescence (IF) and immunohistochemical (IHC) analyses revealed decreased expression of CD31 and Ki-67, respectively, in the combination therapy group, indicating suppressive effects on angiogenesis and tumor cell proliferation (Figure 7F-G). Furthermore, H&E staining of major organs showed no evident pathological lesions in mice treated with luteolin and the BiKE, suggesting that the combination therapy did not cause apparent systemic acute toxicity (Figure 7H).

### Mechanism of the inhibitory effect of luteolin and B7-H3 on NSCLC

**Figure 8:**
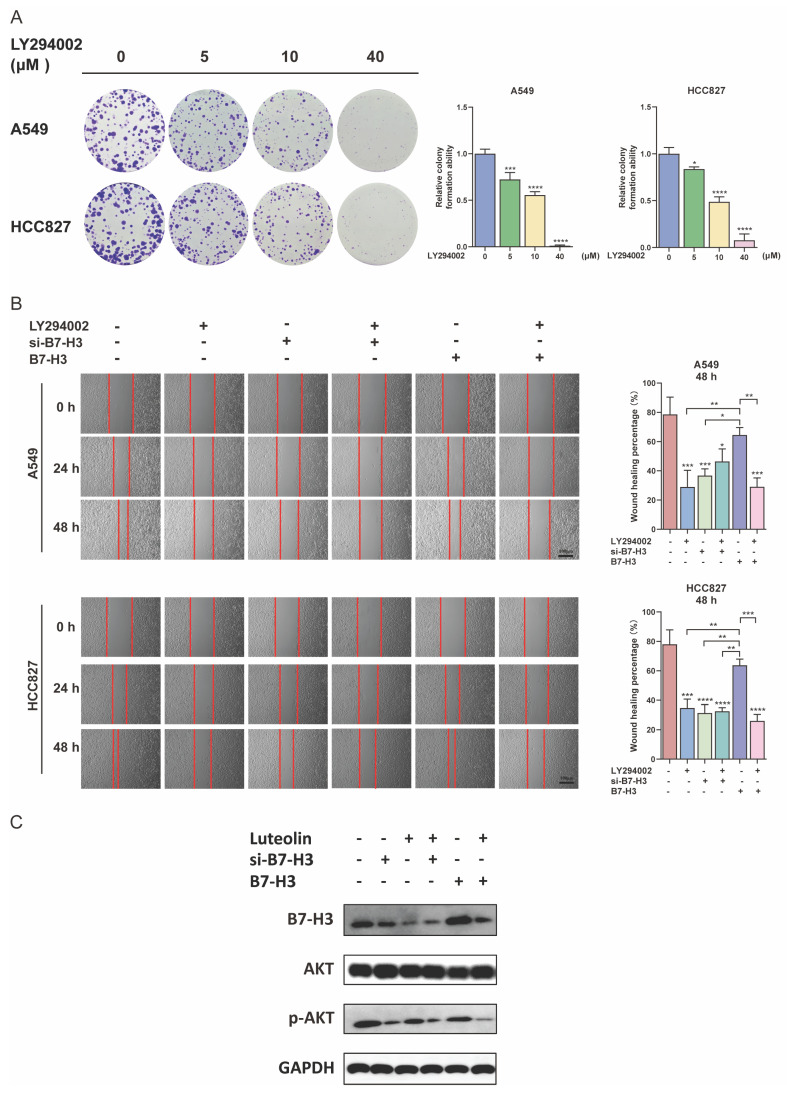
** Effects of luteolin and B7-H3 on NSCLC cells via the PI3K/AKT signaling pathway. (A)** Colony formation of NSCLC cells treated with the indicated doses of the PI3K inhibitor LY294002. **(B)** The migration of NSCLC cells with modulated B7-H3 expression and treated with LY294002 (10 nM). (scale bar=100 μm). **(C)** The influence of B7-H3 and luteolin on AKT and p-AKT levels. *P < 0.05, **P < 0.01, ***P < 0.001, and ****P < 0.0001.

Following these observations of efficacy and safety, we investigated the molecular mechanisms underlying the inhibitory effects of luteolin and B7-H3 knockdown (si-B7-H3) on NSCLC cells. Our bioinformatics analysis suggested an association between B7-H3 and the PI3K/AKT pathway (Figure 1E). Based on this, we confirmed that LY294002, a phosphoinositide 3-kinase (PI3K) inhibitor, dose-dependently inhibited the clonogenic ability in NSCLC cells (Figure 8A). Furthermore, the combination of LY294002 with B7-H3 knockdown resulted in enhanced inhibition of proliferation. Conversely, LY294002 counteracted the growth-promoting effects of B7-H3 overexpression (Figure 8B). This is consistent with what we observed earlier regarding the effect of luteolin and regulating B7-H3 on the proliferation ability of NSCLC cells. Furthermore, we also examined the effect of the combination of LY294002 and luteolin on NSCLC. The results showed that when the two were used together, they could effectively inhibit the proliferation activity and migration ability of NSCLC cells (Figure S5A-B). The changes in protein levels also indicated the regulatory effects when treatments of luteolin as well as the intervention of B7-H3 had an impact on p-AKT levels (Figure 8C). Collectively, these findings demonstrate that the antitumor effects of both luteolin and B7-H3 modulation are mediated, at least in part, through the PI3K/AKT signaling pathway.

## Discussion

Since the widespread application of immunotherapy has led to significant progress in the treatment paradigm of NSCLC, particularly with the approval of anti-PD-1/PD-L1 antibodies, its use is expected to continue increasing in the coming decade 23. Despite these, a subset of NSCLC patients eventually develop resistance to such treatments 24. Moreover, as many patients experience primary or acquired resistance to current immunotherapies, the identification of promising targets and novel immunotherapeutic strategies has become both an unmet need and an opportunity to improve curative outcomes. Therefore, discovering effective combination immunotherapies to enhace clinical efficacy in NSCLC patients remains a priority 25.

As a key player in cancer progression, B7-H3 is preferentially expressed on both tumor cells and immune cells within the tumor microenvironment (TME). The marked difference in its expression between tumor and normal tissues suggests that B7-H3-targeted therapies could achieve cancer-specific efficacy while minimizing damage to healthy cells 26. In addition, studies have established a clear association between B7-H3 expression and poor prognosis. The rapid development of B7-H3-targeted immunotherapies is reflected in the growing number of clinical trials evaluating their safety and efficacy across cancer types. These findings not only support B7-H3 as a promising prognostic biomarker but also encourage the future development of B7-H3-targeting agents for clinical use. The potent anti-proliferative effect observed upon B7-H3 inhibition provides a compelling rationale for prioritizing it in the design of next-generation molecular therapies for NSCLC, potentially benefiting patients with limited treatment options.

Immunotherapies such as chimeric antigen receptor (CAR)-T cells, CAR-NK cells, and PD1/PD-L1 inhibitors have achieved considerable success in clinical practice. However, a proportion of patients still fail to achieve satisfactory therapeutic outcomes 27. Thus, rationally designed combination treatment strategies are highly warranted. Natural products have been reported to influence cancer immunotherapy, primarily by reversing tumor immunosuppression and enhancing immune recognition and therapeutic efficacy. Growing evidence supports the potent anti-tumor effects of flavonoids across various cancers. For instance, myricetin (Myr) alleviated atopic dermatitis symptoms through anti-inflammatory and anti-allergic effects. It also suppressed macrophage-derived chemokine (MDC) and thymus and activation -regulated chemokine (TARC) in interferon-γ (IFN-γ)/tumor necrosis factor-α (TNF-α)-stimulated HaCaT by inhibiting NF-κB and STAT1 signaling pathways 28.

Similarly, rocaglamide (RocA) was shown to promote NK cell infiltration by activating cGAS-STING signaling via induction of mitochondrial DNA (mtDNA) release, establishing it as a potent cGAS-STING agonist with promising potential in cancer immunotherapy, particularly for enhancing NK cell-based strategies 29. Active constituents of taraxacum officinale extract (TOE), namely ICGA-A and CRA, were found to potentiate PD-1 blockade in TNBC. ICGA-A combined with a PD-1/PD-L1 inhibitor enhanced tumor infiltration by macrophages and CD8^+^ T cells while reducing T cell exhaustion. Separately, CRA increased CD8^+^ T cell frequency. Both components also directly suppressed tumor proliferation by inhibiting the FAK/PI3K/AKT/mTOR pathway 30. While the anti-tumor effects of luteolin and B7-H3 targeting have been well documented, the potential combined application between the two and its underlying mechanisms remain incompletely understood. In this study, we evaluated the effects of luteolin on the proliferation of multiple NSCLC cell lines. This combined approach offers a novel strategy to overcome the limitations of monotherapies, potentially leading to more durable and potent treatment outcomes. The enhancement of ADCC by the combination therapy is particularly instructive, suggesting that luteolin may be used to potentiate the efficacy of existing and future BiAb platforms. Preliminary mechanistic insights into apoptosis, ROS, and the PI3K/AKT pathway provide a valuable roadmap for future research, guiding in-depth investigations into the signaling cascades and immunomodulatory effects that underpin this combined application strategy. Such efforts will be crucial for translating these findings into rational combination-based clinical trials.

Regarding the regulatory effects of herbal extracts on immune cells, there have been reports, such as on the flavonoid compound scutellarin, which markedly enhanced the efficacy of immunotherapy with CpG oligodeoxynucleotide (ODN) in the mouse CT26 colon tumor model, with a decrease in tumor-infiltrating regulatory T cells (Tregs) and down-regulation of surface expression of TNFR2 on Tregs. In addition, scutellarin treatment markedly increased IFN-γ-producing CD8^+^ T cells in the TME 31. In this study, we initially discovered and verified the enhanced therapeutic effects of inhibiting B7-H3 and the PI3K/AKT pathway on NSCLC. However, further verification is still needed regarding their specific regulatory mechanisms and sites of action. Although our research has identified that luteolin enhances the efficacy of BiKE, the exact role of luteolin in relation to NK cells still needs to be further investigated and clarified. Furthermore, it remains to be investigated through which specific pathways and mechanisms luteolin regulates NK cells and enhances their therapeutic efficacy against NSCLC. Meanwhile, B7-H3 is a member of the immune checkpoint family, and luteolin may also enhance the therapeutic effect by reducing immune escape. For example, silibinin, a type of flavonoid, has been shown to significantly downregulate PD-L1 expression through the JAK-STAT pathway and increased the immunogenicity of NSCLC 32.

Although we have already demonstrated the enhancement of the therapeutic effect of BiKE on NSCLC by luteolin, there is still much that can be explored. The improved inhibition of NSCLC by the combination of luteolin and BiKE could operate through a coordinated multi-mechanism model. Firstly, luteolin may prime NK cells by enhancing their basal activity and reactivity through modulating key signaling pathways (e.g., MAPK/ERK) and potentially downregulating inhibitory receptors, thereby lowering the activation threshold. This primed state can significantly amplify the CD16-mediated signal and ADCC efficacy triggered by BiKE's targeted engagement between NK and tumor cells. Secondly, luteolin could counteract the immunosuppressive TME, notably by inhibiting the TGF-β pathway 33, which is beneficial for enhancing the function of NK cells. Finally, the robust NK cell activation leads to enhanced direct cytotoxicity and substantial secretion of inflammatory factors like IFN-γ and TNF-α. This not only directly kills tumor cells but also recruits and activates broader adaptive immunity, collectively overcoming immunosuppression and tumor heterogeneity. In addition, the clinical application of such herbal medicine-immunotherapy combinations faces significant challenges, primarily concerning unpredictable toxicity and potential induction of immune tolerance. To address toxicity and complex interactions, rigorous pharmacokinetic studies and phased toxicity profiling in relevant preclinical models are essential. The pharmacokinetic profile of luteolin is characterized by extensive phase II metabolism. Due to the action of phenolsulfonyl transferase in the intestine, its main metabolite in human plasma is luteolin-3'-sulfate. Studies indicate that only a small fraction of orally administered luteolin is excreted unchanged in urine (6.6%) and feces (31.3%), suggesting extensive biotransformation. Metabolites including glucuronides, sulfate conjugates, and methylated forms have been detected in both rat and human plasma, as well as in tissues such as the liver, kidney, and small intestine. These metabolites may enter systemic circulation or undergo enterohepatic recycling, collectively contributing to luteolin's biological effects* in vivo* 34. Employing advanced analytics to standardize herbal extracts and identifying their active immunomodulatory constituents can ensure batch-to-batch consistency and clarify mechanisms. Furthermore, well-designed clinical trials that incorporate biomarker-driven patient stratification are crucial to objectively assess efficacy, monitor for immune suppression, and validate safety, thereby paving the way for informed regulatory evaluation.

However, the polypharmacological profile of luteolin necessitates careful consideration for interpreting our mechanistic findings, as the observed enhancement of NK cell-mediated cytotoxicity and application with B7-H3-targeted therapy may stem not only from the proposed mechanisms but also from unrecognized off-target interactions. In addition, while the absence of histopathological changes or significant accumulation in major organs, as determined by immunohistochemistry, suggests a favorable safety profile for luteolin, this does not entirely preclude potential off-target effects. The analytical focus on tissue structure and accumulation may not capture subtle functional or molecular interactions, such as transient binding to enzymes like cytochromes P450, modulation of various kinase signaling pathways, or phytoestrogenic activities, which could occur without causing overt tissue damage. A more comprehensive risk assessment would therefore require sensitive molecular techniques and longer-term studies to fully elucidate luteolin's off-target potential. For therapeutic translation, while luteolin's multi-target nature raises concerns about potential unpredictable toxicities in clinical applications, it may simultaneously offer advantages in overcoming resistance to highly specific targeted therapies. To advance its clinical potential, systematic characterization of luteolin's target landscape through chemoproteomic approaches and development of structurally optimized derivatives with improved selectivity will be essential for designing safe and effective combination immunotherapy regimens.

In summary, this study establishes a novel therapeutic paradigm by combining the natural flavonoid luteolin with B7-H3-targeted immunotherapy for NSCLC. Firstly, we validated B7-H3 as a promising immunotherapeutic target in this context. Subsequently, we demonstrated that the combination of luteolin with a B7-H3-targeted BiKE yields superior antitumor efficacy compared to either agent alone. Mechanistically, this enhanced effect is achieved through a dual-action approach: on one hand, luteolin as well as B7-H3 inhibition suppresses tumors by inducing apoptosis, elevating ROS levels, and modulating the PI3K/AKT pathway; on the other hand, luteolin concurrently potentiates B7-H3-targeted NK cell-mediated cytotoxicity, thereby enabling effective BiKE therapy. These findings collectively define a novel and promising therapeutic strategy for NSCLC, which combines the natural flavonoid luteolin with B7-H3-targeted immunotherapy. This work may provide valuable insights and a foundation for future clinical applications and related research.

## Supplementary Material

Supplementary figures.
