# Supplementary material for: Luteolin enhances antitumor immunity of B7-H3-targeted bispecific natural killer cell engagers against non-small cell lung cancer
Source: Int J Biol Sci. 2026 Mar 25;22(7):3731–48. doi: 10.7150/ijbs.125834 (PMC13086014; doi:10.7150/ijbs.125834)
Supplement: Supplementary file 1 — Supplementary figures. [file ijbsv22p3731s1.pdf]

Supplementary Figures

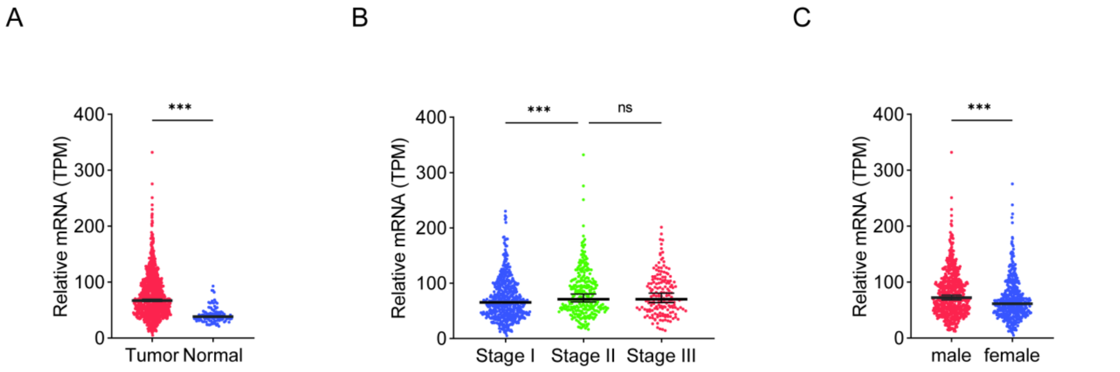

Figure S1. Expression landscape of CD276 (B7-H3) in NSCLC. (A) B7-H3 expression was markedly upregulated in NSCLC tumors compared to normal lung tissues. **(B)** Expression levels demonstrated a positive correlation with disease stage, showing a significant increase from stage II to stage I. **(C)** A significant association was found between gender and B7-H3 expression, suggesting it may be a relevant factor in disease biology.

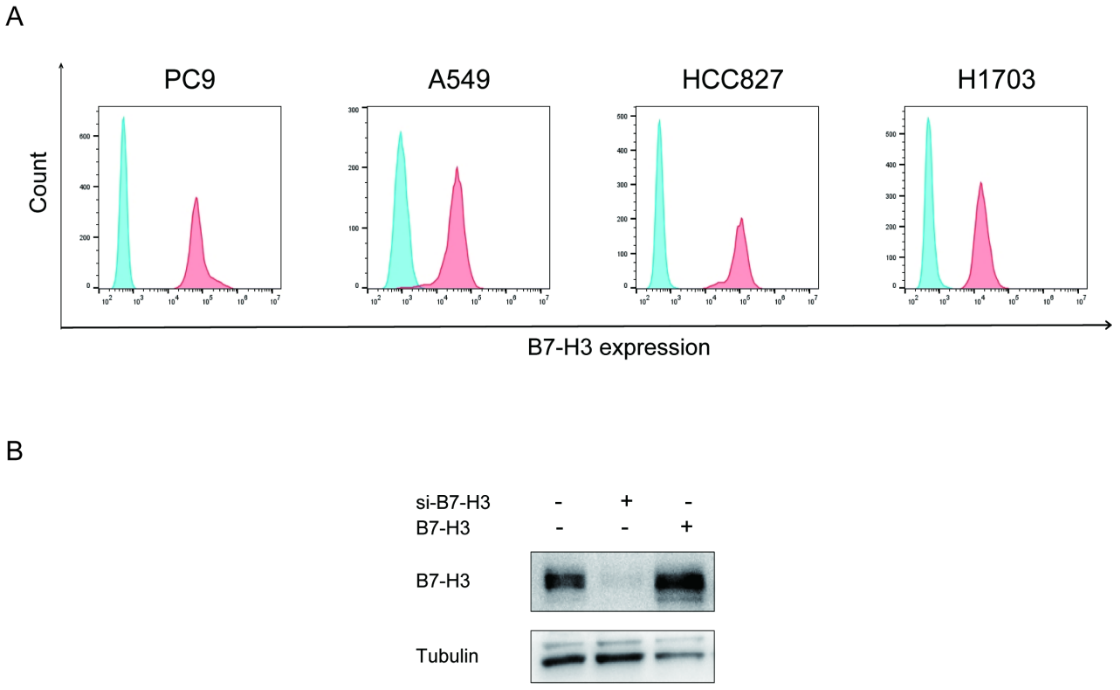

Figure S2. Detection of B7-H3 expression. (A) Flow cytometric analysis of B7-H3 surface expression in NSCLC cells lines. **(B)** B7-H3 expression was verified by Western blotting.

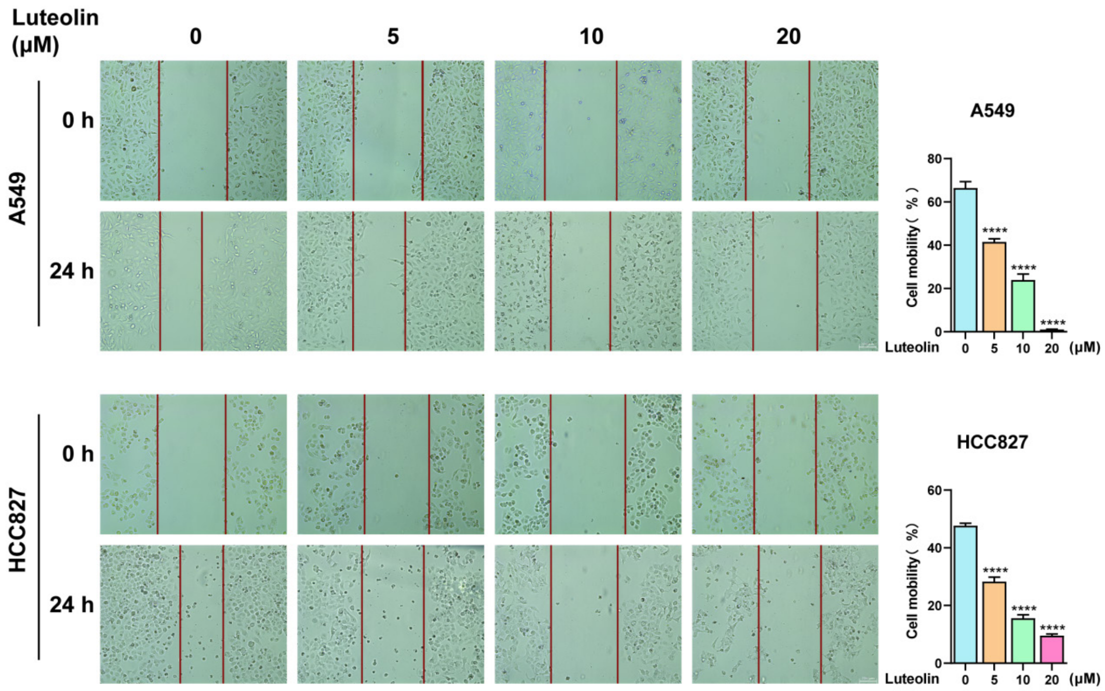

Figure S3. Migration of luteolin-treated NSCLC cells assessed by wound-healing assay. Representative images of A549 and HCC827 cell migration are shown (scale bar=200 μm). ****P < 0.0001.

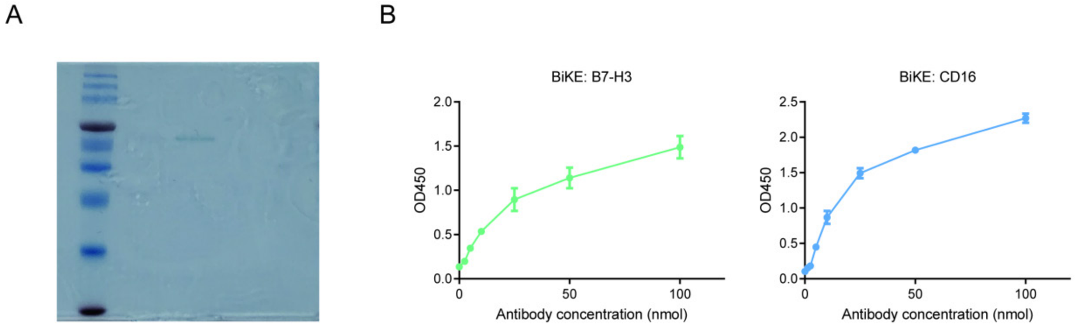

Figure S4. Expression and binding analysis of B7-H3/CD16 bispecific antibody. (A) Purified B7-H3/CD16 bispecific antibody. **(B)** Binding capacity of the B7-H3/CD16 bispecific antibody to B7-H3 and CD16 antigen, respectively.

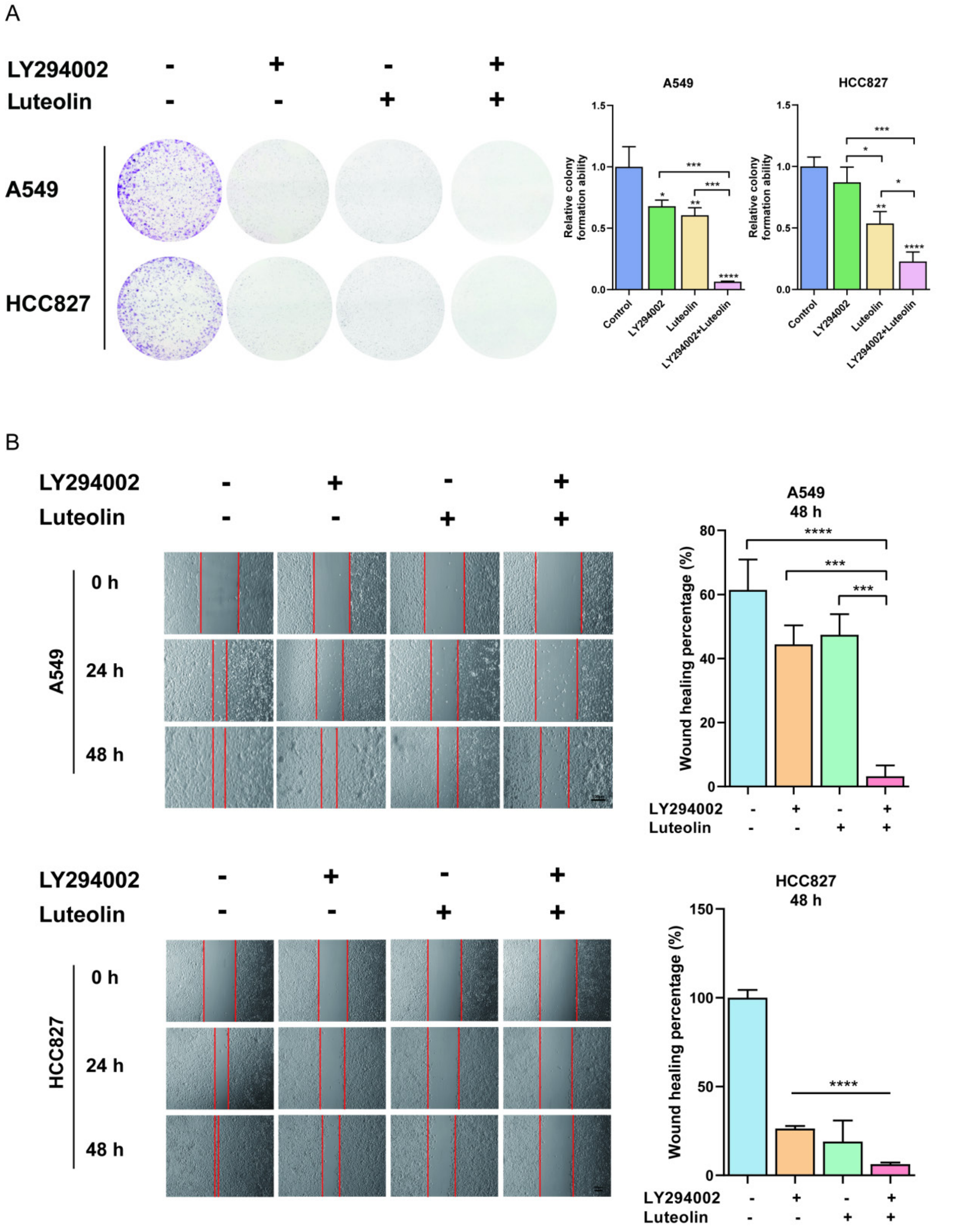

Figure S5. Effects of LY294002 and luteolin on NSCLC cells. (A) Colony formation of NSCLC cells treated with either LY294002 or luteolin alone or in combination. **(B)** The migration of NSCLC cells with either LY294002 or luteolin alone or in combination. (scale bar=100 μm). The concentrations of LY294002 and luteolin are 10 nM, respectively. *$P < 0.05$, **$P < 0.01$, ***$P < 0.001$, and ****$P < 0.0001$.
